# Gender Differences in Interpersonal Coping and Depressive Symptoms During Emerging Adulthood

**DOI:** 10.3390/bs16050682

**Published:** 2026-04-30

**Authors:** Chong Man Chow, Caitlyn Thelen, Sarah Dean, Ellen Hart

**Affiliations:** 1 Psychology Department, Eastern Michigan University, Ypsilanti, MI 48197, USA; 2 Psychology Department, University of Wisconsin-Oshkosh, Oshkosh, WI 54901, USA; 3 Psychology Department, Radford University, Radford, VA 24142, USA

**Keywords:** interpersonal coping, depressive symptoms, gender, emerging adulthood, parent, peer

## Abstract

The current study examined how interpersonal coping styles (anxious/expressive, dismissive, and adaptive) within parent and peer domains were associated with depressive symptoms in emerging adults, and whether these associations were moderated by gender. The sample included 352 undergraduate students at a Midwestern university (41% women, 59% men), primarily White/Caucasian (88.9%), with a mean age of 18.91 years (*SD* = 0.97). Participants completed the Interpersonal Coping Styles Questionnaire and the Brief Symptom Inventory. At the bivariate level, anxious/expressive coping with both parents and peers were associated with higher depressive symptoms, whereas adaptive coping was not significantly related to depressive symptoms. Dismissive coping was associated with depressive symptoms only in the parent domain. In regression analyses, peer-related coping accounted for additional variance in depressive symptoms beyond parent-related coping, whereas the reverse was not observed. Gender moderated several associations. Among men, anxious/expressive coping with peers was positively associated with depressive symptoms, whereas adaptive coping with peers was negatively associated. Among women, dismissive coping with peers was associated with higher depressive symptoms. These findings highlight the relevance of relational context and gender in understanding depressive symptoms.

## 1. Gender Differences in Interpersonal Coping and Depressive Symptoms During Emerging Adulthood

Emerging adulthood (ages 18–25) is marked by substantial transition and instability, as many individuals leave home, complete their education, begin careers, and in some cases form long-term romantic partnerships or start families (Arnett, 2000). For many, this period is experienced as growth-promoting, with evidence suggesting improvements in well-being and mental health across the transition (Galambos et al., 2006). At the same time, emerging adulthood is also associated with heightened vulnerability. Approximately 40% of individuals in this age range meet criteria for at least one mental health disorder, representing a higher prevalence than any other adult age group (Kessler et al., 2005). Mood disorders are particularly common, affecting an estimated 12.9% of emerging adults. Given that emerging adulthood is characterized by increased autonomy, identity exploration, and developmental flexibility (Arnett, 2005), it represents a critical window for intervention. Understanding how emerging adults’ interpersonal coping strategies are associated with depressive symptoms can deepen theoretical insight into psychopathology during this developmental stage and inform prevention and intervention efforts aimed at strengthening resilience and reducing symptom severity.

### 1.1. Interpersonal Coping Styles

The coping literature is extensive but has historically emphasized individual-level strategies, with comparatively less attention to how partners cope together. A formal model of dyadic coping emerged more recently (Bodenmann, 2005; Randall & Bodenmann, 2009), conceptualizing coping within relationships as a process in which one partner communicates stress and seeks support, followed by a positive or negative response from the other partner (Chow et al., 2014). Although this framework advanced the field, limitations remain, including a reliance on one partner’s perceptions of support rather than the support actually provided, as well as limited consideration of the broader interpersonal dynamics that shape coping exchanges (Chow et al., 2014). To address these gaps, Chow et al. (2014) integrated attachment theory (Bowlby, 1982; Cassidy, 1994) and self-determination theory (Ryan et al., 2005) with the dyadic coping framework, proposing three interpersonal coping styles: dismissive, anxious/expressive, and adaptive. Together, these three interpersonal coping styles provide a theoretically integrated framework for understanding how attachment processes, motivational regulation, and relational dynamics jointly shape how partners cope with stress within close relationships.

The dismissive interpersonal coping style is characterized by low support-seeking, strong self-reliance, and suppression of attachment needs. In stressful situations, individuals with this style tend to manage difficulties independently and are unlikely to reach out to their partner for assistance (Chow et al., 2014). Conceptually, this style draws from avoidant attachment in attachment theory and suppressive forms of regulation in self-determination theory. Within the broader coping literature, it aligns most closely with avoidance coping (Endler & Parker, 1990), which involves disengaging from or ignoring stressors rather than confronting them directly.

The anxious/expressive interpersonal coping style involves excessive reassurance-seeking, difficulty coping independently, and challenges in emotion regulation. Individuals with this style rely heavily on their partner during times of stress and may become overly dependent on relational support. This conceptualization reflects anxious attachment—marked by heightened concerns about closeness and separation—and parallels forms of emotional dysregulation described in self-determination theory. In traditional coping frameworks, anxious/expressive coping resembles emotion-focused coping, including venting, rumination, reassurance-seeking, and efforts to reinterpret stressors (Baker & Berenbaum, 2007; Lazarus & Folkman, 1984).

The adaptive interpersonal coping style reflects a balanced integration of self-reliance and support-seeking. Individuals with this style effectively regulate their own emotions while also engaging their partner constructively to manage stress. This style is conceptually grounded in secure attachment and emotional integration within self-determination theory. In the coping literature, it most closely resembles problem-focused coping, which involves actively addressing stressors through strategies such as generating solutions, weighing alternatives, and implementing concrete steps toward resolution (Baker & Berenbaum, 2007; Lazarus & Folkman, 1984).

### 1.2. Coping and Depressive Symptoms

A substantial body of research links coping styles to depressive symptoms. In general, problem-focused coping has been associated with better psychological adjustment, including fewer depressive symptoms (Aldao et al., 2010; Endler & Parker, 1994; Nolen-Hoeksema & Aldao, 2011). In contrast, findings for emotion-focused and avoidance coping have been more complex and, at times, inconsistent. One source of inconsistency is definitional overlap. Emotion-focused coping encompasses a broad range of strategies, and in some work (e.g., Baker & Berenbaum, 2007), avoidance has been conceptualized as a subtype of emotion-focused coping, making it difficult to disentangle their unique effects. Moreover, not all forms of emotion-focused coping are uniformly maladaptive. For example, Baker and Berenbaum (2007) found that emotional processing—a potentially adaptive form of emotion-focused coping—was associated with increased positive affect among men but not women. Nonetheless, across studies, emotion-focused and avoidance-oriented coping have more consistently been linked to greater depressive symptoms (Aldao et al., 2010; Baker & Berenbaum, 2007; Endler & Parker, 1990; Nolen-Hoeksema & Aldao, 2011).

Although Chow et al. (2014) demonstrated that dismissive, anxious/expressive, and adaptive interpersonal coping styles are associated with relationship quality (e.g., intimacy, conflict), their links to broader psychological adjustment remain underexplored. Drawing from the broader coping literature, avoidance- and emotion-oriented styles are generally associated with higher depressive symptoms, whereas task-oriented (i.e., problem-focused) coping is associated with fewer symptoms (Smith et al., 2016). Extending this logic to the interpersonal domain, it is reasonable to hypothesize that dismissive and anxious/expressive interpersonal coping styles will be positively associated with depressive symptoms, whereas the adaptive interpersonal coping style will be negatively associated with depressive symptoms.

### 1.3. Gender Differences

Epidemiological research documents gender differences in depressive symptoms, with women reporting higher rates of depressive disorders than men in the general population (American Psychiatric Association, 2013; Parker & Brotchie, 2010). Similar patterns have been observed during emerging adulthood (Parker & Brotchie, 2010). One explanation attributes these differences to gender socialization processes, whereby men and women are encouraged to regulate emotions in distinct ways (Nolen-Hoeksema, 2012). From this perspective, gender differences in depressive symptoms may be mediated by emotion regulation or coping strategies. However, empirical findings provide only partial support for this account. Research suggests that men are more likely to engage in avoidant coping (e.g., escape or distraction), whereas women are more likely to use emotion-focused strategies such as rumination (Mainiero, 1986; Pearlin & Schooler, 1978; Rosario et al., 1988; Sigmon et al., 1995). Yet both avoidant and emotion-focused coping have been linked to greater depressive and anxiety symptoms (Nolen-Hoeksema, 2012). If both gender-typical coping styles are associated with elevated symptoms, a straightforward mediation model—where gender predicts coping, which predicts depressive symptoms—becomes less compelling.

Instead, we propose that the fit between gender role expectations and coping styles may be more consequential than the specific coping strategy. Deviating from gender norms can elicit social disapproval, exclusion, or isolation (Mulvey & Killen, 2015), potentially heightening distress and discouraging coping behaviors perceived as gender-incongruent. Supporting this perspective, prior work has shown that gender can moderate the association between emotion-focused coping and internalizing symptoms (Kelly et al., 2008). Thus, we hypothesize that individuals who adopt coping styles incongruent with gender role expectations will report greater depressive symptoms than those whose coping aligns with gender norms, due to the added strain of norm violation.

Consistent with prior findings, men report greater use of dismissive interpersonal coping, whereas women report greater use of anxious/expressive interpersonal coping; no gender differences were observed for adaptive coping (Chow et al., 2014). Building on this evidence, we anticipate that men who engage in anxious/expressive coping and women who engage in dismissive coping will report elevated depressive symptoms. In contrast, we do not expect gender to moderate the association between adaptive coping and depressive symptoms, given its normatively acceptable nature. Testing these hypotheses may clarify how coping processes intersect with gender norms to shape depressive risk and offer a more nuanced account of gender disparities in depressive symptoms during emerging adulthood.

### 1.4. Parents Versus Peers in Emerging Adulthood

During emerging adulthood, increasing independence from parents is a central developmental task (Arnett, 2015b). Yet, despite physical and psychological separation, many emerging adults continue to rely on parental support—financially, emotionally, and practically (Arnett, 2015a). Because this period involves shifting reliance from parents to peers (including close friends and romantic partners), alongside elevated rates of mood disorders, it represents a particularly important window for examining how these relational contexts contribute to depressive symptoms.

As emerging adults establish autonomy, some scholars argue that friendships and romantic relationships become especially salient, at times even surpassing family relationships in perceived importance (Robnett & Leaper, 2013). High-quality friendships have been associated with greater self-worth and fewer internalizing symptoms (Barry et al., 2009; Pittman & Richmond, 2008). Moreover, peers are often viewed as central in shaping identity development and decision-making during this stage (Fromme et al., 2008; Hopmeyer & Medovoy, 2017; Ohannessian et al., 2017; Roisman et al., 2004).

At the same time, evidence continues to support the enduring influence of parents on well-being in emerging adulthood. Strong parent–child relationships are associated with higher life satisfaction and smoother adjustment to adult roles, particularly when parental support is frequent or perceived as meaningful (Fingerman et al., 2012). Similarly, Inguglia et al. (2014) found that greater perceived closeness and support from parents were linked to higher levels of well-being. Interestingly, with developmental shifts during this period, maladaptive forms of parental involvement may be associated with internalizing difficulties, such as depressive and anxiety symptoms (Angelini et al., 2025).

Overall, the literature presents a mixed picture regarding whether parents or peers exert greater influence on emerging adults’ mental health. Notably, Pettit et al. (2011) directly compared perceived social support from parents and peers in predicting depressive symptoms and found that parental support—but not peer support—significantly predicted depressive symptoms. Drawing on this work and the broader literature, we hypothesize that interpersonal coping within both parental and peer relationships will significantly predict depressive symptoms. Given the mixed findings in the literature, we do not propose specific differential predictions regarding whether parents or peers will account for greater variance in the outcome. Rather, we examine whether each domain explains unique variance in depressive symptoms above and beyond the other.

### 1.5. The Current Study

In this study, we had three primary research goals. First, we sought to examine whether specific interpersonal coping styles were associated with depressive symptoms in emerging adults. We predicted that the adaptive interpersonal coping style would be associated with fewer depressive symptoms, whereas maladaptive interpersonal coping styles (i.e., anxious/expressive coping and dismissive coping) would be associated with higher levels of depressive symptoms (Hypothesis 1).

Second, we examined whether interpersonal coping with parents and with peers would be associated with depressive symptoms. We predicted that coping with both parents and peers would be significantly related to depressive symptoms in emerging adults (Hypothesis 2).

Third, we examined whether gender would moderate the association between interpersonal coping and depressive symptoms. We predicted that the fit, or lack of fit, between gender and coping style would be related to individuals’ depressive symptoms (Hypothesis 3). Specifically, we expected that women who utilize dismissive interpersonal coping would report more depressive symptoms than women who utilize adaptive or anxious/expressive coping with parents or peers. Similarly, we expected that men who utilize anxious/expressive coping would report more depressive symptoms than men who utilize adaptive or dismissive interpersonal coping.

## 2. Methods

### 2.1. Participants and Procedures

Participants consisted of a non-clinical sample of 352 undergraduate students from a Midwestern university. The sample included 145 women (41%) and 207 men (59%). Participants identified as White/Caucasian (88.9%), African American/Black (3.7%), Asian (4.3%), Hispanic/Native American (1.7%), and Other (1.4%). The mean age of participants was 18.91 years (*SD* = 0.97).

Students enrolled in the university’s SONA research participation system signed up for the study through that platform. They completed a series of questionnaires either online or via a paper-and-pencil format. All participants received course credit toward a research participation requirement in an undergraduate psychology class. Participants were exclusively psychology students who participated to fulfill course requirements.

For the online version, participants were instructed to complete the survey at a location of their choosing, ensuring a quiet and private environment. For the paper-and-pencil version, participants signed up for designated time slots and completed the survey in small groups under the supervision of a graduate assistant. Privacy was maintained during these group sessions.

The current research protocol was approved by the University of Wisconsin–Oshkosh IRB. For both procedures, informed consent was obtained prior to completing the questionnaires. Participation was entirely voluntary, and participants could withdraw from the study at any time without penalty.

### 2.2. Measures

#### 2.2.1. Interpersonal Coping

The Interpersonal Coping Styles Questionnaire (ICSQ) is a 15-item self-report questionnaire that measures how individuals communicate and share their stress with close others (Chow et al., 2014). The ICSQ uses a 5-point Likert scale from 1 (*not at all accurate/descriptive*) to 5 (*very accurate/descriptive*). The ICSQ consists of three subscales which measure three different styles of interpersonal coping: dismissive coping, anxious/expressive coping, and adaptive coping. Participants were instructed to reflect on how they typically involve their target relationships when coping with stress, excluding stressors related to the person or the relationship. The dismissive style reflects suppression of attachment needs and self-reliance (e.g., “I want to deal with things on my own rather than depending on my _____ for help”), the adaptive style reflects effective emotion regulation and use of partner support (e.g., “I try to solve my own problems but will also go to my _____ for advice”), and the anxious/expressive style reflects excessive reassurance seeking (e.g., “I talk to my _____ over and over to find relief”). For this study, participants completed the items separately for each relationship target (father, mother, best friend, and romantic partner). They were instructed that parent-like figures may be used when referring to mothers or fathers. For romantic partner items, participants who were not currently in a relationship are asked to respond based on a past or hypothetical relationship. For the purposes of the present study, scores for mothers and fathers were combined to form a parent interpersonal coping composite, whereas scores for best friends and romantic partners were combined to form a peer interpersonal coping composite. This measure has shown good reliability with Cronbach’s alpha values ranging from 0.82 to 0.88 for the three subscales (Chow et al., 2014). In this study, we found Cronbach’s alpha values ranging from 0.70 to 0.84 for the three subscales, across four domains. The average Cronbach’s alpha values were 0.76 for the dismissive coping subscale, 0.82 for the adaptive coping subscale, and 0.77 for the anxious/expressive coping subscale.

#### 2.2.2. Depressive Symptoms

Participants completed the 6-item depressive symptoms subscale of the Brief Symptom Inventory (BSI; Derogatis & Melisaratos, 1983). Participants read a list of problems and complaints and decided how often they are bothered or distressed by that problem (e.g., “Feeling no interest in things.”) on a scale ranging from 1 (almost never) to 4 (almost always). For this study, the reliability was excellent (Cronbach’s α = 0.85).

### 2.3. Planned Analyses

Prior to model testing, we computed descriptive statistics including means, standard deviations, and correlations of the studied variables. Hypothesis 1 was examined primarily using bivariate correlations.

For Hypothesis 2, a series of hierarchical linear regression analyses were conducted to examine whether interpersonal coping with parents and peers was associated with depressive symptoms. Age, gender, and method of data collection (paper-and-pencil vs. Qualtrics online administration) were included as covariates in all regression analyses to account for potential demographic differences and data collection method-related variance that may be associated with depressive symptoms and reporting of interpersonal coping. Specifically, separate regression models were estimated to examine the contributions of interpersonal coping with parents and interpersonal coping with peers in explaining depressive symptoms. These analyses were intended to estimate the associations between coping dimensions within each relational context (parents vs. peers) and depressive symptoms. Next, a combined model was estimated in which interpersonal coping with parents and peers were entered simultaneously. This model allowed for the examination of the relative contributions of coping with parents versus peers, providing a more stringent test of whether each set of coping dimensions was uniquely associated with depressive symptoms when controlling for the other. In addition, this model served as the basis for comparison with the parent-only and peer-only models, allowing for the evaluation of the extent to which each set of variables accounted for unique variance in depressive symptoms (Δ*R*^2^) above and beyond their shared variance.

Finally, moderation analyses were conducted to test whether student gender moderated the associations between interpersonal coping strategies and depressive symptoms. Interaction terms between gender and each coping dimension were created (after appropriate centering of continuous variables) and entered in a subsequent step of the regression model. A significant increase in explained variance (Δ*R*^2^), along with significant interaction terms, was interpreted as evidence of moderation.

## 3. Results

Means, standard deviations, and correlations among the study variables are presented in Table 1.

### 3.1. Hypotheses 1 and 2

Hypothesis 1 predicted that adaptive interpersonal coping would be associated with fewer depressive symptoms, whereas maladaptive coping styles (i.e., anxious/expressive and dismissive coping) would be associated with higher levels of depressive symptoms. These hypotheses were examined using bivariate correlations (see Table 1) and were partially supported. Specifically, anxious/expressive interpersonal coping with both parents and peers was significantly associated with higher depressive symptoms. In contrast, adaptive interpersonal coping with parents and peers was not significantly related to depressive symptoms. For dismissive coping, only coping with parents was significantly associated with depressive symptoms, whereas dismissive coping with peers was not.

For Hypothesis 2, we conducted a series of hierarchical linear regression analyses to examine whether interpersonal coping with parents and peers were associated with depressive symptoms (see Table 2). We first estimated separate models for each relational domain. Model 1, which included interpersonal coping with parents, accounted for a significant proportion of variance in depressive symptoms (*R*^2^ = 0.08, *p* < 0.001). Model 2, which included interpersonal coping with peers, also accounted for a significant proportion of variance (*R*^2^ = 0.13, *p* < 0.001).

When both parent and peer interpersonal coping variables were entered simultaneously (Model 3), the overall model remained significant (*R*^2^ = 0.13, *p* < 0.001). Comparing Model 3 to Model 1 indicated that peer interpersonal coping explained an additional 5% of the variance in depressive symptoms beyond parent coping (Δ*R*^2^ = 0.05, *p* < 0.001). In contrast, comparing Model 3 to Model 2 showed that parent interpersonal coping did not account for additional variance beyond peer coping (Δ*R*^2^ = 0.01, *p* = 0.51).

Examination of the regression coefficients in Model 3 revealed that peer interpersonal coping was a significant correlate of depressive symptoms. Specifically, adaptive coping with peers was negatively associated with depressive symptoms, whereas anxious/expressive coping with peers was positively associated. Notably, adaptive peer coping was not significantly related to depressive symptoms at the bivariate level; thus, its emergence in the regression model should be interpreted cautiously, as it may reflect a suppression effect. In contrast, none of the parent interpersonal coping variables were significant correlates in Model 3.

### 3.2. Hypothesis 3

**Figure 1:**
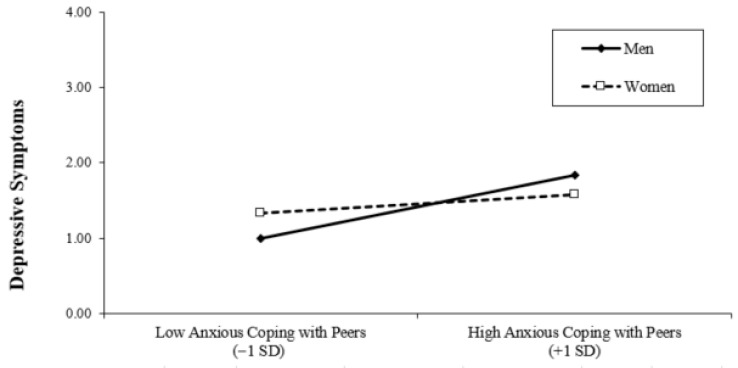
Moderating Effect of Gender on Anxious/Expressive Coping with Peers.

**Figure 2:**
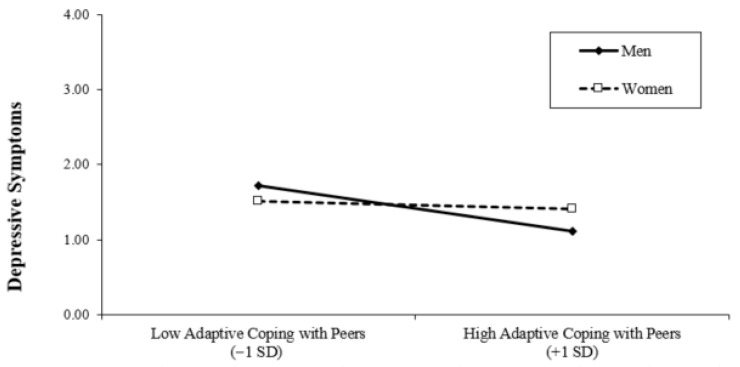
Moderating Effect of Gender on Adaptive Coping with Peers.

**Figure 3:**
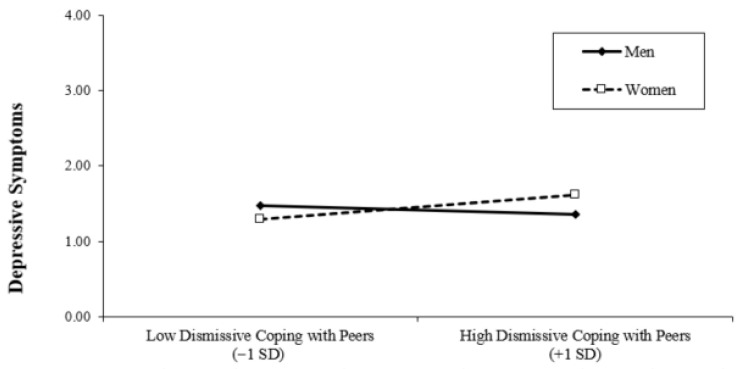
Moderating Effect of Gender on Dismissive Coping with Peers.

Finally, we examined whether student gender moderated the associations between interpersonal coping strategies and depressive symptoms. Interaction terms between gender and each coping dimension were created (after appropriate centering of continuous predictors) and entered into the regression model. To facilitate interpretation of the interaction effects, we estimated the same model twice using different reference codings of gender. In Model 4 (men), gender was coded as 0 = men and 1 = women, such that the regression coefficients for the coping variables represent simple slopes for men. In Model 4 (women), gender was recoded as 0 = women and 1 = men, allowing the coefficients to be interpreted as simple slopes for women. These two models are statistically equivalent and differ only in the reference group used for interpretation. The inclusion of interaction terms resulted in a significant increase in explained variance (Δ*R*^2^ = 0.04, *p* = 0.03), indicating that the associations between interpersonal coping and depressive symptoms differ by gender. Examination of the simple slopes showed that among men, higher engagement in anxious/expressive coping with peers was associated with higher depressive symptoms (see Figure 1), whereas adaptive coping with peers was associated with lower depressive symptoms (see Figure 2). In contrast, these associations were not significant among women. Also, among women, higher engagement in dismissive coping with peers was associated with higher depressive symptoms, and such an association was not significant among men (see Figure 3). Gender did not significantly moderate the association between interpersonal coping with parents and depressive symptoms.

## 4. Discussion

Our first hypothesis was that adaptive interpersonal coping would be associated with fewer depressive symptoms, whereas anxious/expressive and dismissive coping would be associated with higher depressive symptoms. This hypothesis was partially supported based on the bivariate correlations. Specifically, anxious/expressive coping with both parents and peers was significantly associated with higher depressive symptoms. In contrast, adaptive interpersonal coping with both parents and peers was not significantly related to depressive symptoms. For dismissive coping, only coping with parents was significantly (albeit modestly) associated with higher depressive symptoms, whereas dismissive coping with peers was not significantly related to depressive symptoms.

These findings provide partial support for the broader coping literature. The positive associations between anxious/expressive coping and depressive symptoms are consistent with prior research linking emotion-focused coping to greater psychological distress (e.g., Aldao et al., 2010; Baker & Berenbaum, 2007; Endler & Parker, 1990; Nolen-Hoeksema & Aldao, 2011). However, the absence of significant associations between adaptive interpersonal coping and depressive symptoms contrasts with studies suggesting that problem-focused coping is related to fewer depressive symptoms (e.g., Aldao et al., 2010; Endler & Parker, 1994). Importantly, the lack of significant bivariate associations for some coping dimensions should be interpreted with caution, as these patterns may reflect underlying gender-related or cultural differences in coping processes. These possibilities will be examined more closely in the moderation analyses discussed later.

Our second hypothesis proposed that interpersonal coping with both parents and peers would each show independent associations with depressive symptoms above and beyond the other relational domain. This hypothesis was only partially supported. When examined separately, both parent-based coping (Model 1) and peer-based coping (Model 2) accounted for significant variance in depressive symptoms, although the magnitude of these effects was modest. However, when both sets of predictors were entered simultaneously (Model 3), only peer-based coping accounted for unique variance in depressive symptoms, whereas parent-based coping did not. Thus, the hypothesis that both domains would demonstrate independent associations above and beyond one another was only partially supported. Instead, peer-based coping accounted for additional variance in depressive symptoms beyond parent-based coping, whereas parent-based coping did not account for additional variance beyond peer-based coping. This pattern is consistent with prior work suggesting that peers may play a prominent role, along with parents, during emerging adulthood (e.g., Barry et al., 2009; Fromme et al., 2008; Hopmeyer & Medovoy, 2017; Pittman & Richmond, 2008; Robnett & Leaper, 2013; Roisman et al., 2004), and contrasts with findings reported by Pettit et al. (2011). At the same time, given the modest amount of variance explained, these findings should be interpreted cautiously. Rather than concluding that peers are more important than parents, it is more accurate to suggest that peer-related coping showed a stronger unique association with depressive symptoms after accounting for shared variance between relational domains.

The associations between interpersonal coping with peers and depressive symptoms were moderated by gender, supporting our third hypothesis. Specifically, the pattern of associations differed for men and women. Among men, higher engagement in anxious/expressive coping with peers was associated with greater depressive symptoms, whereas adaptive coping with peers was associated with lower depressive symptoms. In contrast, these associations were not significant among women. For women, higher engagement in dismissive coping with peers was associated with greater depressive symptoms, a pattern not observed among men.

These findings are broadly consistent with the expectation that coping strategies less aligned with gendered norms (e.g., anxious/expressive coping for men and dismissive coping for women) may be linked to greater psychological distress. Prior research suggests that gender shapes associations between coping styles and depressive symptoms (e.g., Kelly et al., 2008), and these results align with perspectives emphasizing the role of gender norms in psychological adjustment (e.g., Mulvey & Killen, 2015). However, it is important to note that the current study did not directly assess gender role beliefs or conformity to gender norms, and thus conclusions regarding underlying mechanisms should be interpreted cautiously.

Women and men are often socialized to manage emotions differently, particularly in interpersonal contexts (Nolen-Hoeksema, 2012; Rose & Rudolph, 2006). From a social-cognitive perspective, women and girls tend to adopt a more relational orientation, placing greater emphasis on interpersonal relationships and investing more heavily in them (Rose & Rudolph, 2006). Consistent with this, prior work has shown that such orientations are associated with greater engagement in co-rumination among girls compared to boys (Rose, 2002), which has been linked to increased risk for internalizing symptoms.

Against this backdrop, the present findings are somewhat unexpected. Anxious/expressive coping—conceptually similar to co-rumination—was not associated with depressive symptoms among women, but was positively associated among men. This pattern suggests that the role of anxious/expressive coping may extend beyond a simple mediational account (i.e., gender differences in coping explaining gender differences in distress). Instead, it points to differential associations depending on gender. This interpretation is consistent with evidence showing that emotion-focused coping is associated with higher distress among men but lower distress among women (Hamid et al., 2023).

A similar gender-specific pattern emerged for dismissive coping, which was associated with higher depressive symptoms among women but not men. Together, these findings suggest that, in addition to gender differences in the use of coping strategies, the degree to which coping aligns with or deviates from gendered expectations may have implications for psychological adjustment (Mulvey & Killen, 2015).

Interestingly, adaptive coping was associated with lower depressive symptoms among men but not women. Adaptive interpersonal coping reflects a flexible and effective use of social support, often considered a “healthy” coping strategy. Although women generally report greater use of social support than men (Tamres et al., 2002), these findings suggest that men may derive particular benefit when they do engage in adaptive interpersonal coping. This highlights that support-seeking is not simply a matter of frequency, but also of quality. While both anxious/expressive and adaptive coping reflect an approach-oriented tendency toward others, they differ qualitatively—one emphasizing emotionally intense, potentially co-ruminative exchanges, and the other reflecting flexible and constructive engagement with social resources.

Finally, although the discussion above frames depressive symptoms as the outcome, the cross-sectional nature of the data precludes causal conclusions. It is also possible that psychological maladjustment influences coping patterns and their alignment with gender norms. Moreover, the overall model accounted for a modest proportion of variance in depressive symptoms (*R*^2^ = 0.17), indicating that these moderation effects, while statistically significant, should be interpreted with appropriate caution.

Our findings contribute to the existing literature in several ways. First, our results are consistent with prior research suggesting that peers may play a relatively salient role in the lives of emerging adults compared to parents (Fromme et al., 2008; Hopmeyer & Medovoy, 2017; Ohannessian et al., 2017; Robnett & Leaper, 2013; Roisman et al., 2004). Importantly, this pattern reflects differences in associations observed in the present study and should not be interpreted as evidence of causal influence. Second, this study extends the coping literature by examining a relatively understudied construct and measure, the Interpersonal Coping Styles Questionnaire (ICSQ; Chow et al., 2014). Interpersonal coping, as conceptualized by Chow et al. (2014), remains underexplored, and the present findings provide preliminary evidence regarding its associations with depressive symptoms, while also highlighting its relatively limited explanatory power in this context. More broadly, these findings contribute to the literature on coping and psychological adjustment by illustrating that the associations between coping strategies and depressive symptoms may vary across relational contexts. Although the overall predictive power was modest, the observed patterns are consistent with the possibility that socialization processes, including gender-related expectations, may be relevant to understanding how coping strategies are linked to depressive symptoms.

### 4.1. Clinical Relevance

The results of this study add to the understanding of some of the correlates of depressive symptoms in emerging adults, a population with a high vulnerability to clinical depression. We found that, when collapsing across gender and relationship domains, anxious/expressive interpersonal coping appears to be the most consistent correlate of higher depressive symptoms. This suggests that, although seeking social support has long been regarded as an adaptive way of dealing with stress, excessive reliance on others for support may be associated with poorer psychological adjustment. Thus, these findings highlight a potentially important area for intervention, in which emerging adults may benefit from developing greater self-efficacy in coping, which prior research has linked to better stress management. Additionally, our results suggest that friends and romantic partners may have relatively stronger associations with emerging adults’ depressive symptoms than parents. Clinicians working with emerging adults with depressive symptoms may consider assessing the quality and function of peer and romantic relationships, as this may be helpful in explaining depressive symptoms. However, because interpersonal coping with peers accounted for a small but significantly larger amount of variance, clinicians should not ignore clients’ relationships with their parents. We also found that gender moderated the associations between peer-domain interpersonal coping and depressive symptoms. For clinicians, these findings suggest that intervention efforts may benefit from considering how coping operates within the context of gender role expectations, as approaches that do not account for these differences may be less effective.

### 4.2. Limitations and Future Directions

Several limitations should be noted. First, all data were collected from a single source—the participant. Interpersonal coping was assessed solely from the individual’s perspective, and questionnaires were not administered to parents, peers, or romantic partners. Because prior research using the ICSQ (Chow et al., 2014) has employed dyadic designs, relying on one informant may limit the accuracy of classifying coping styles within relationships. Future studies should incorporate dyadic methods to obtain reports from both partners, which may provide a more comprehensive understanding of adaptive, anxious/expressive, and dismissive coping patterns. Second, the sample limits generalizability. Participants were undergraduate students at a four-year university, predominantly White (88%), and relatively young (M age = 18.91). Findings may not generalize to non-college emerging adults, more racially and ethnically diverse populations, or individuals further along in this developmental period. For instance, the college context itself may shape interpersonal coping processes, as students often experience social environments that differ from those of non-college emerging adults. These contextual differences may also add complexity to how social relationships are associated with psychological adjustment and how these associations vary by gender. Furthermore, although the sample included more men than women, this imbalance was modest and both groups were sufficiently represented to permit the moderation analyses conducted. Future research should include more diverse samples in terms of educational background, race/ethnicity, and age to better evaluate developmental and contextual influences. Third, the cross-sectional, correlational design prevents conclusions about directionality. It remains unclear whether interpersonal coping styles contribute to depressive symptoms or whether depressive symptoms influence coping behaviors. Longitudinal research is needed to clarify temporal ordering. Fourth, although we examined gender as a moderator, the variable used in this study reflects a binary classification of men and women and does not capture broader aspects of gender identity, gender role socialization, or individuals’ endorsement of gender norms. As a result, our interpretation of the findings in terms of “fit” or incongruence with gender role expectations remains speculative. Future research would benefit from incorporating more nuanced and multidimensional measures of gender, including gender identity and gender role beliefs, to more directly examine the mechanisms underlying these associations.

## 5. Conclusions

In sum, this study highlights the role of interpersonal coping—particularly within peer and romantic relationships—in understanding depressive symptoms during emerging adulthood. These findings underscore the importance of considering relational contexts and gendered coping processes when examining depressive symptoms in this period and point to meaningful directions for future research and intervention. Future research should employ longitudinal or dyadic designs to better clarify the directionality of associations among gender, interpersonal coping, and depressive symptoms in emerging adulthood.

## Figures and Tables

**Table 1:** Means, Standard Deviations, and Correlations of Study Variables.

Variable	1	2	3	4	5	6	7
1. Dismissive Coping—Parent	--						
2. Adaptive Coping—Parent	−0.34 *						
3. Anxious Coping—Parent	−0.31 *	0.46 *					
4. Dismissive Coping—Peers	0.59 *	−0.01	−0.05				
5. Adaptive Coping—Peers	−0.03	0.42 *	0.19 *	−0.11 *			
6. Anxious Coping—Peers	0.00	0.06	0.54 *	−0.15 *	0.48 *		
7. Depressive Symptoms	0.11 *	−0.02	0.19 *	0.07	0.02	0.28 *	--
Mean (Men)	3.24	3.10	2.31	2.80	3.26	2.70	1.83
*SD*	0.87	0.80	0.84	0.81	0.78	0.95	0.65
Mean (Women)	3.00	3.46	2.68	2.55	3.81	3.11	1.97
*SD*	0.97	0.88	0.80	0.78	0.71	0.76	0.60

*Note.* * *p* < 0.05.

**Table 2:** Hierarchical Regression Analyses.

	Model 1	Model 2	Model 3	Model 4 (Men)	Model 4 (Women)
Variable	*B* (SE)	*B* (SE)	*B* (SE)	*B* (SE)	*B* (SE)
Data (0 = pencil, 1 = online)	−0.09 (0.07)	−0.12 (0.07)	−0.11 (0.07)	−0.10 (0.07)	−0.10 (0.07)
Gender	0.12 (0.07)	0.13 (0.07)	0.14 (0.07)	0.14 (0.48)	−0.14 (0.48)
Age Dismissive w/Parent	−0.01 (0.03) 0.11 (0.04) *	−0.01 (0.03)	−0.01 (0.03) 0.07 (0.05)	−0.02 (0.03) 0.14 (0.08)	−0.02 (0.03) 0.00 (0.06)
Adaptive w/Parent	−0.07 (0.05)		0.05 (0.05)	0.13 (0.08)	−0.04 (0.07)
Anxious w/Parent	0.18 (0.05) *		−0.01 (0.06)	−0.10 (0.10)	0.08 (0.08)
Dismissive w/Peers		0.09 (0.04) *	0.04 (0.05)	−0.06 (0.08)	0.16 (0.07) *
Adaptive w/Peers		−0.14 (0.05) *	−0.17 (0.06) *	−0.31 (0.10) *	−0.06 (0.08)
Anxious w/Peers		0.26 (0.04) *	0.26 (0.06) *	0.42 (0.09) *	0.12 (0.08)
Dismissive w/Parent × Gender				−0.14 (0.10)	0.14 (0.10)
Adaptive w/Parent × Gender				−0.18 (0.11)	0.18 (0.11)
Anxious w/Parent × Gender				0.18 (0.13)	−0.18 (0.13)
Dismissive w/Peers × Gender				0.21 (0.11) *	−0.21 (0.11) *
Adaptive w/Peers × Gender				0.25 (0.12) *	−0.25 (0.12) *
Anxious w/Peers × Gender				−0.31 (0.12) *	0.31 (0.12) *
Multiple *R*^2^	0.08	0.13	0.13	0.17	0.17
F-Statistic (*df*)	5.011 (6, 339) *	8.16 (6, 339) *	5.58 (9, 336) *	4.47 (15, 330) *	4.47 (15, 330) *

*Note.* * *p* < 0.05. *B* = unstandardized regression coefficients, with standard errors in parentheses. Δ*R*^2^ (M3–M1) = 0.05, *p* < 0.001; Δ*R*^2^ (M3–M2) = 0.01, *p* = 0.51; Δ*R*^2^ (M4–M3) = 0.04, *p* = 0.03. In Model 1 to Model 4 (men), Gender was coded as 0 = M, 1 = W, while in Model 4 (women), Gender was coded as 0 = W, 1 = M. These results suggest that interpersonal coping variables—particularly peer-related coping and their interactions with sex—contribute small to moderate associations with depressive symptoms.

## Data Availability

The datasets in the current study are available from the corresponding author on reasonable request. The procedure of data sharing will require the IRB’s approval from both the corresponding author’s and the requester’s institutions.
